# Rapid Quantification of SARS-CoV-2 Neutralising Antibodies Using Time-Resolved Fluorescence Immunoassay

**DOI:** 10.3390/vaccines10122149

**Published:** 2022-12-15

**Authors:** Gary R. McLean, Yueke Zhang, Rene Ndoyi, Adam Martin, Julian Winer

**Affiliations:** 1 School of Human Sciences, London Metropolitan University, London N7 8DB, UK; 2 National Heart and Lung Institute, Imperial College London, London W2 1PG, UK; 3 PremaLabs Diagnostics UK Ltd., London W1J 6ER, UK; 4 Winer Clinic London, London W1G 7DB, UK

**Keywords:** SARS-CoV-2, neutralising antibodies, point-of-care diagnostic test

## Abstract

The quantification of neutralising antibodies (NAb) for SARS-CoV-2 has become an important tool for monitoring protective immunity following infection or immunisation. In this study, we evaluated using World-Health-Organisation-standard immunoglobulin preparations, a novel point-of-care test that quantitates NAb by time-resolved fluorescent immunoassay. The assay provided robust data of binding antibody units (BAU) in 15 min that were well correlated with NAb values obtained by traditional in vitro neutralisation assay. The data also correlated well to spike-receptor-binding domain-binding antibodies over a broad range of plasma dilutions. The assay was extremely sensitive, able to detect positive samples after dilution 1:10,000 and over a wide range of BAU. Assay specificity was estimated at 96% using Pre-COVID-19 serum samples when applying a cut-off value of 47 BAU/mL, although readings of up to 100 BAU/mL could be considered borderline. This point-of-care diagnostic test is useful for rapid population screening and includes the use of capillary blood samples. Furthermore, it provides results for SARS-CoV-2 NAb in 15 min, which can inform immediate decisions regarding protective immunity levels and the need for continued COVID immunisations.

## 1. Introduction

The emergence and subsequent spread of severe acute respiratory syndrome coronavirus (SARS-CoV-2) from late 2019 was responsible for the worldwide COVID-19 pandemic [1]. This has resulted in the rapid development of a plethora of diagnostic tests for the virus [2] and specific antibodies [3], including the immunological response to vaccines [4] and neutralising antibodies (NAb) [5].

**Figure 1:**
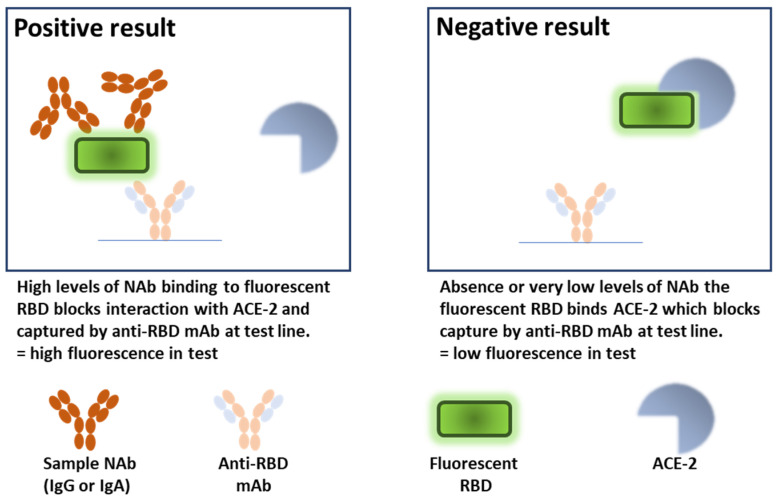
Schematic overview of PL-NAb test function. NAb in test sample bind to fluorescent SARS-CoV-2-RBD that is captured at the test line by specific mAb, causing a fluorescence signal proportional to level of NAb to be detected (**left panel**). In the absence of NAb within samples, the fluorescent RBD molecule is free to bind to ACE-2. This interaction blocks the capture at the test line, resulting in a low fluorescent signal (**right panel**). Graphics are not drawn to scale.

At this current stage of the COVID-19 pandemic, population-level knowledge of protective immunity is necessary to establish vaccine efficacy and the proportion of individuals at risk from infection or re-infection with SARS-CoV-2. Although it is desirable to determine both cell-mediated and humoral protective immunity, the latter is comparatively technically easier to perform. However, traditional assays for the detection of SARS-CoV-2 NAb, such as the plaque reduction neutralisation test (PRNT), require assays in which protective antibodies in samples are detected using live virus and the infection of mammalian cells [6]. These assays require several days to complete, skilled operators, and the use of BSL3 laboratories. Assays using pseudo-typed virus (pVNT) are effective and overcome the safety requirement [7] but do not address the time issues, nor are they high-throughput enough for mass testing. More recently, protein-based binding methods without infectious virus and cells have been developed to detect Nab, allowing for simplicity and speed over the time-consuming in vitro authentic virus-neutralising assays [8]. These tests, known as surrogate virus neutralisation tests (sVNT), detect NAb via chemiluminescence or enzymatic readouts of interference between the SARS-CoV-2 spike (S) receptor binding domain (RBD) and the entry receptor angiotensin-converting enzyme (ACE-2) [9]. The sVNT provide good agreement with PRNT and pVNT. In addition, sVNT are well suited to large-scale population testing, even though the PRNT and, to some extent, the pVNT remain the gold-standard assays. Numerous diagnostic tests have been applied throughout the pandemic and have offered benefits at various stages. It is clear now, with a largely immune population, that large-scale detection of NAb levels would be the most useful diagnostic method (for review, see [10]). A study evaluating a direct comparison of vaccine-induced NAb by PRNT and pVNT amongst performing laboratories revealed significant discrepancies that could be alleviated with the use of World Health Organisation (WHO)-standard immunoglobulins [11]. Thus, standardisation and simplification of NAb testing methods is critical for establishing immune correlates of protection, which is a key factor that can influence vaccine development. The large-scale use of sVNT is one approach which could lead to the adoption of NAb as a surrogate endpoint for vaccine efficacy that could streamline future COVID-19 vaccine efforts systematically on a global scale. One such sVNT, the PremaLabs Diagnostics SARS-CoV-2 NAb test kit, is a rapid in vitro immunochromatographic test (Time-Resolved Dry Fluorescence Immunoassay, TRFIA) for the qualitative detection of NAb to SARS-CoV-2 in human serum, plasma, or whole blood. The test kit serves as an aid in identifying individuals with adaptive immune responses to SARS-CoV-2, which are indicative of past infection or successful immunisation. The test cassette device is similar to a standard lateral flow device for detecting IgG to SARS-CoV-2 [12] and is made up of two precoated lines: test and control. The area at the test line is coated with monoclonal anti-RBD antibody. During the test, SARS-CoV-2-specific NAb in the sample interact with recombinant RBD conjugated to fluorescent particles, forming an antibody–antigen–fluorescent particle complex that migrates by capillary action on the membrane until it reaches the test line. The complex is bound by the capture antibodies on the test line to develop time-resolved fluorescence proportional to the amount of NAb in the sample. For a negative reaction, the recombinant RBD (without bound NAb) can bind to the free recombinant ACE-2 receptor proteins, blocking the binding site of capture antibody at the test line, resulting in low fluorescence (see Figure 1).

The aim of the current study was to evaluate the performance of the PremaLabs Diagnostics COVID-19 rapid NAb test (PL-NAb) to determine its sensitivity using SARS-CoV-2 reference immunoglobulins from the World Health Organisation (WHO) and specificity using Pre-COVID-19 serum samples that would be negative for NAb.

## 2. Materials and Methods

### 2.1. Samples and Standards

National Institute for Biological Standards and Control (NIBSC)-standard immunoglobulins were obtained from the Medicines and Healthcare products Regulatory Agency (MHRA). These reagents were the first WHO international reference panel for anti-SARS-CoV-2, developed in December 2020. The panel contained pooled plasma samples obtained from individuals that had recovered from COVID-19 and a negative control plasma obtained from healthy blood donors before 2019. The immunoglobulins panel, previously evaluated by a WHO international collaborative study [13], contained 5 individual components: NIBSC code 20/150 (high); 20/148 (mid); 20/144 (low anti-S, high anti-N); 20/140 (low); and 20/142 (negative).

Pre-COVID-19 serum samples from a prior study performed at Imperial College London [14] were used to define the range of negative values and determine assay specificity. Ethical approval had been obtained previously with informed consent.

### 2.2. Enzyme-Linked Immunosorbent Assay (ELISA)

Indirect ELISA was performed to determine the binding specificity of NIBSC standards to SARS-CoV-2 recombinant spike S1 and nucleocapsid (N) antigens (Cambridge Bioscience, Cambridge, UK). This analysis was designed to establish relative levels of S- and N-binding immunoglobulins contained within the NIBSC plasma preparations. We performed ELISA as described previously [15], with minor modifications. SARS-CoV-2 antigens were diluted to 1 μg/mL in PBS and coated into ELISA plate wells. Plasma was serially titrated 2-fold, beginning at 1:100 and ending at 1:51,200 using PBS containing between 20 and 5% milk powder (PBSTM). Bound antibodies were detected using anti-human IgG or anti-human IgA HRP conjugates (Southern Biotechnology Associates, Birmingham, AL, USA) diluted 1:1000 in PBSTM. Reactions were developed using TMB substrate (Thermo Fisher Scientific, Waltham, MA, USA) before obtaining optical density at 450 nm.

### 2.3. SARS-CoV-2-Neutralising Antibody Diagnostic Test (PL-NAb)

The method was followed in accordance with the manufacturer’s instructions. Human plasma or serum samples were diluted where necessary in sterile PBS. An amount of 20 μL of sample (triplicate determinations) was added to 80 μL of the supplied sample diluent, mixed, and added to the test strip device. After 15 min incubation, dry fluorescence was measured using the LS-1100 (medi) or the LS-21100 (maxi) dry fluorescence analyser (Lansion Biotechnology Co., Ltd., Nanjing, China). Data were generated in BAU/mL with a detection range of 10–3000. Measurements >40 BAU/mL are considered a positive result based on prior analysis of 150 negative samples. According to the manufacturers, the positive test rate of SARS-CoV-2 NAb for 50 confirmed cases was (46/50) 92.00%. The negative coincidence rate of SARS-CoV-2 NAb for 150 negative samples was (143/150) 95.33%.

### 2.4. Data Graphics and Analysis

Data obtained were plotted using GraphPad Prism version 9.4.1 (GraphPad Software, LLC, San Diego, USA). Data were first evaluated for normal distribution to ascertain appropriate statistical tests and linear regression was assessed by Spearman’s correlation and the r values stated.

## 3. Results

We evaluated the performance of the PremaLabs Diagnostics COVID-19 rapid NAb (PL-NAb) test (https://premalabsdiagnostics.com/covid-19-nab-test/ accessed on 23 September 2022) using the NIBSC standard immunoglobulins, which formed the first World Health Organisation (WHO) international reference panel for anti-SARS-CoV-2. The immunoglobulin panel contains pooled plasma samples obtained from individuals recovered from COVID-19 and a negative control plasma obtained from healthy blood donors before 2019. These had previously been evaluated by a WHO international collaborative study and harmonised against live virus neutralisation assays for NAb, anti-RBD IgG, anti-S1 IgG, anti-Spike IgG, and anti-N IgG (Table 1) [13]. The panel’s five individual components were therefore assigned as high, mid, low anti-S and high anti-N, low, and negative for SARS-CoV-2 antibodies. Thus, individual components contained a range of values recorded as IU/mL for NAb (relative to the International Standard 20/136) and the standard BAU/mL for binding antibodies. Larger neutralising antibody values were contained in the high component (1473 IU/mL), with decreasing values seen as mid (210 IU/mL), low S high N (65 IU/mL), and low (44 IU/mL). The negative component was consistently very low or undetectable (Table 1). Values for binding antibodies to spike, receptor binding domain (RBD), spike S1 subunit (S1), and nucleocapsid (N) showed similar trends, with the high component containing the largest BAU/mL and decreasing values seen with the other plasma components (Table 1).

**Figure 2:**
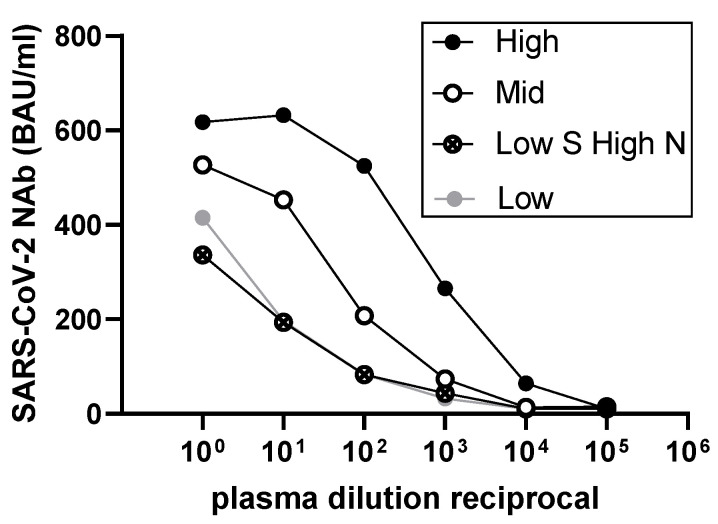
NIBSC standards display exceptional linearity when diluted and analysed via TRFIA. High, mid, low S high N, and low NIBSC standards were titrated in 10-fold steps and analysed for NAb using the PL-NAb test. Data are displayed as BAU/mL versus dilution and are represented by three independent determinations.

Our aim was to use the NIBSC standards to determine the linearity, sensitivity, and specificity of the PremaLabs Diagnostic NAb test (referred to as PL-NAb). Serial 10-fold dilution of the four NIBSC standards (high, mid, low S high N, and low) that have binding activity was performed to determine linearity of the NAb content using PL-NAb. This analysis used the LS-1100 device and time-resolved fluorescence immunoassay (TRFIA) to determine antibody interference with SARS-CoV-2 S-RBD (original Wuhan strain accession number YP_009724390.1) binding to the entry receptor angiotensin-converting enzyme (ACE-2), which is represented as BAU/mL. Figure 2 shows that the four NIBSC standards show reproducible linearity of NAb detection over the titration range, with detection still apparent at plasma dilutions of 1:1000. Saturation of the high NIBSC sample was observed at dilutions lower than 1:10, which was not observed with other samples. At the lower end of detection, the high NIBSC standard still gave a positive detectable result (64 BAU/mL) when diluted 1:10,000, whereas other samples at this dilution were below the limit of detection (LOD) of 10 BAU/mL. Importantly, these PL-NAb data are consistent with data obtained using our ELISA binding assay, which was calibrated using the NIBSC standards (Table 2). Endpoint titration (first plasma dilution to become <0.2 OD450 nm) of the NIBSC standards and binding analyses by ELISA demonstrated that the high NIBSC standard contained the highest level of IgG and IgA to SARS-CoV-2 S and is followed sequentially with reducing levels of binding found with mid, low S high N, and low NIBSC standards. IgG- and IgA-binding capacity to SARS-CoV-2 N follows similar trends, although this determinant is not detected by the PL-NAb test. Therefore, we established using ELISA that the NIBSC standards show similar trends, in terms of binding to S (Table 2), to those that we observe with surrogate neutralization using PL-Nab (Figure 2).

**Figure 3:**
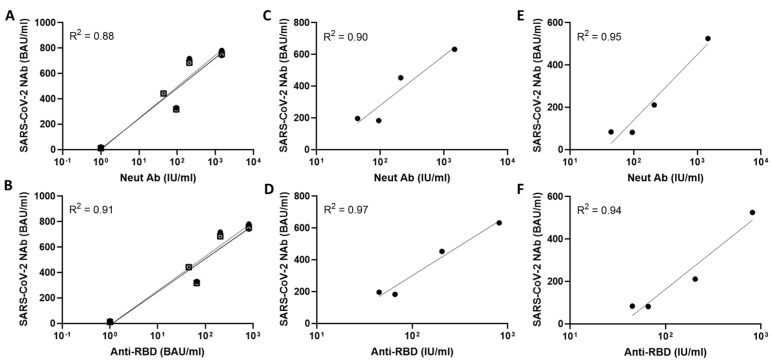
Correlations of levels of NIBSC standards NAb to SARS-CoV-2 by TRFIA with WHO data Neut Ab (**A**,**C**,**E**) and Anti-RBD (**B**,**D**,**F**). (**A**,**B**) represent undiluted NIBSC standards; (**C**,**D**) represent 1:10 dilution of NIBSC standards; and (**E**,**F**) represent 1:100 dilution of NIBSC standards. Correlations by linear regression are shown as R^2^ values. Data are shown as the average of 3 independent analyses.

Next, we correlated these NAb data obtained with the NIBSC standards to the reported titre of NAb (IU/mL) and anti-RBD (BAU/mL). Excellent correlations were observed with undiluted plasma (Figure 3A,B), plasma diluted 1:10 (Figure 3C,D), and plasma diluted 1:100 (Figure 3E,F). Correlations were consistently greater than R^2^ of 0.90 when analysing the NAb data, except with undiluted plasma samples including the NIBSC negative when correlated with WHO values for NAb (R^2^ = 0.88). Overall, the correlations observed were similar throughout, but tended to be stronger, such that greater R^2^ values were seen when comparing PL-NAb data with anti-RBD antibody rather than NAb. These data suggest that the NAb test faithfully reproduces neutralisation data generated by PRNT with excellent sensitivity, and that anti-RBD antibodies in plasma are the likely neutralising component detected.

**Figure 4:**
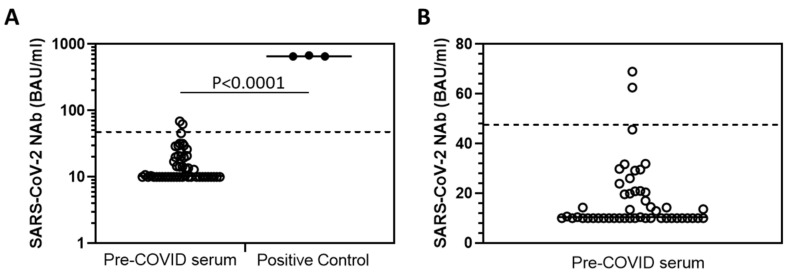
Scatterplots of NAb determination by TRFIA of Pre-COVID-19 serum samples (triplicate) to determine specificity and positive/negative cut-off value (dotted horizontal line = 47.58 BAU/mL). (**A**): displays scatter of Pre-COVID-19 serum data with respect to positive control (high NIBSC standard), significantly different *p* < 0.0001. (**B**): displays scatter range in values of Pre-COVID-19 serum data alone.

Lastly, we determined the PL-NAb assay specificity using 50 serum samples from pre-COVID-19 volunteers from a prior study [14]. Undiluted serum was analysed via a PL-NAb test and the LS-21100 analyser to produce scatter plots and determine the positive/negative cut-off value for BAU/mL that reflects functional NAb. Pre-COVID-19 serum samples produced mostly very low BAU/mL values at the LOD of 10 BAU/mL (Figure 4). Sample values ranged from the LOD to 68.99 BAU/mL, with a mean of 15.45 BAU/mL (standard deviation 16.07). Applying the mean + 2SD rule to these data provides a positive/negative cut-off value of 47.58 BAU/mL (dotted lines Figure 4). Out of the 50 samples, two were marginally above this cut-off, reflecting an assay specificity of 96%, which is consistent with prior observations that estimate the cut-off value to be 40 BAU/mL with 95.45% specificity (according to documents provided by the test manufacturers).

## 4. Discussion

Here, we have evaluated the performance of a new quantitative diagnostic test for SARS-CoV-2 NAb. The test is rapid; requires just 20 μL of plasma, serum, or whole blood; and provides a quantitative measure of the level of NAb in 15 min, with excellent similarity to traditional methods of detecting SARS-CoV-2 NAb. The test can therefore faithfully reflect levels of protective immunity to SARS-CoV-2 and could be a useful population screening tool to inform decisions regarding continued immunisation. Our analysis relied on the use of a set of standard immunoglobulin preparations promoted by the WHO as reagents that allow a standardised international reference panel to evaluate SARS-CoV-2 antibody diagnostics across regions and different tests. We also included a set of Pre-COVID-19 serum samples to estimate the diagnostic cut-off point at which a reliable measure of potential protective NAb could be expected.

The PL-NAb diagnostic test is a competitive immunoassay where the specific SARS-CoV-2 NAb inhibit the binding of the recombinant SARS-CoV-2 RBD, which has been fluorescently labelled, to recombinant human ACE-2. Detection is achieved by dry fluorescence in a time-resolved manner (TRFIA) following UV excitation of chelated lanthanide immunomicrospheres [16]. The assay allows for a stronger signal intensity to be detected at a slightly later timepoint compared with traditional immunofluorescence and improves the quality and accuracy of detection by minimising background emission interference. The data output determines NAb according to binding antibody units, a normalised measure that is based on the WHO standard, enabling comparisons to be made between different tests. The analytical measuring range is reported to be from 10 to 3000 BAU/mL, reflecting a significant breadth of detection spanning the likely sample variations. Linearity of the PL-NAb test was determined across the analytical measuring range using NIBSC standard immunoglobulins containing different levels of SARS-CoV-2 neutralising and binding antibodies. We did not observe BAU/mL >1000 with any of the standards, reflecting the levels of NAb in individuals that had recovered from a single SARS-CoV-2 infection early in the pandemic, rather than having received multiple immunisations with COVID-19 vaccines. Furthermore, at the upper detection range, saturation of detection was noted when assaying an undiluted plasma sample containing high levels of SARS-CoV-2 antibodies (NIBSC high). This phenomenon was not evident with immunoglobulin preparations containing lower levels of antibodies or with more dilute plasma. In fact, linearity of NAb detection was exceptional at these lower levels, reflecting good assay sensitivity and a low potential for false negatives. Importantly, we confirmed these data obtained with PL-Nab using alternative ELISA binding experiments.

Correlation of the PL-NAb test with NAb and RBD binding, according to the published WHO data for these preparations, was found to be very high. This reflects the fact that the majority of NAb to SARS-CoV-2 will interact with the RBD [17]. Of course, the assay will not determine other sites of NAb interaction with spike, such as those binding to the N-terminal domain (NTD), but these are likely to be of minimal contribution compared to RBD-binding NAb in the neutralising effect on SARS-CoV-2 [18]. For this reason, we did not correlate PL-NAb data with the other parameters for the standards supplied by NIBSC, such as anti-S1, anti-S and anti-N. However, inspecting the WHO data shown in Table 1 and based on our own analyses shown in Figure 3, it is likely that similar correlations to those we observed for NAb and RBD binding would be observed. Comparisons of antibody units, such as IU/mL for NAb and BAU/mL for binding, have been established, but are difficult to compare directly, particularly when different assays are used to define these parameters. However, it has been proposed that 1000 BAU/mL can be used to compare assays, particularly for the same class of immunoglobulin (e.g., IgG or IgA) and their specificity (e.g., S or N) [19]. Since the PL-NAb test determines NAb binding to RBD as a surrogate for neutralising, the use of BAU/mL values can be considered an appropriate measure to reflect the level of protective antibodies in a sample and therefore the level of protection afforded against SARS-CoV-2 in vivo. In fact, levels of anti-S IgG >2000 BAU/mL are routinely observed in samples measured using PL-NAb from fully vaccinated and COVID-19-experienced individuals. Approximately 90% of adults (age range 18–80) recorded levels above 1000 BAU/mL. Therefore, 1000 BAU/mL could be considered a benchmark for a decision regarding the timing of further immunisation against SARS-CoV-2 [20]. Despite a current lack of consensus on a robust immune correlate of protection for SARS-CoV-2, evidence does suggest that anti-S and NAb is a likely readout. However, translation of this to BAU/mL currently remains elusive, although values of 154 BAU/mL anti-S for ancestral SARS-CoV-2 and 171 BAU/mL anti-S for the alpha variant have been recorded as thresholds of protection [21], suggesting that 1000 BAU/mL with PL-Nab represents exceptional protection.

The lower BAU/mL values for NAb seen at assay lower LOD are more difficult to link to protective capabilities. Low positive or borderline samples have been classified as having SARS-CoV-2 S antibody levels <100 BAU/mL [22]. Our analysis of diluted NIBSC standards revealed that even the low standards produced BAU/mL values >100 when diluted 1:100, well below the dilution factor when analysing whole blood samples. The high NIBSC standard required dilution to 1:10,000 to reduce BAU/mL to <100, reflecting good assay sensitivity at the lower end of detection. Translation of this value to in vivo protection is not entirely possible, as it is just one component of protective immunity that is more complex, including a cell-mediated component [23,24]. Nevertheless, we could determine a lower-level cut-off of approximately 48 BAU/mL when analysing the range of values obtained using Pre-COVID-19 serum samples. Thus, one could envisage that the PL-NAb test would have the following conditions assigned based on currently available information.
**PL-NAb (BAU/mL)****Outcome**0–50Negative for NAb51–100Borderline or low positive for NAb (not protected)101–1000Moderately protective immunity>1000Fully protected (in terms of SARS-CoV-2 disease severity)

## 5. Conclusions

The PL-NAb test is a rapid and accurate alternative to PRNT for determining levels of NAb to SARS-CoV-2. It can assist with determinations of protective levels of immunity to SARS-CoV-2 at a population level and can assist with decisions regarding the further need for booster immunisations or not. The test does not provide information about the full spectrum of immunity and may require updating as new variants with altered RBD sequences emerge. However, it offers a snapshot of an essential protective immunity component that reflects immune status to this relatively new virus. We now know that having immunity to SARS-CoV-2 from prior infection or immunisation results in improved outcomes following infection, including reduced symptoms and disease severity and lower hospitalisation and death rates, even as new variants emerge [25]. At this late stage of the COVID-19 pandemic, when we are living with COVID-19, determinations of SARS-CoV-2 NAb levels at a population level could go some way towards allowing a return to pre-pandemic behaviour, speeding up the transition to a post-pandemic world based on immune protection status.

## Figures and Tables

**Table 1:** Anti-SARS-CoV-2 antibody titres are calculated as the geometric mean of the potencies obtained from the collaborative study participants calibrated against the WHO International Standard for anti-SARS-CoV-2 immunoglobulin (NIBSC 20/136). The five components are shown at the top with corresponding NIBSC code in brackets. Neutralising antibody (Neut Ab), anti-receptor binding domain (anti-RBD), anti-spike 1 subunit (Anti-S1), anti-spike (Anti-S1/S2), anti-nucleocapsid (Anti-N) rows are listed. IU: International Units; BAU: binding antibody units; n.d.: not detected.

	High (20/150)	Mid (20/148)	Low S, High N (20/144)	Low (20/140)	Negative (20/142)
Neut Ab (IU/mL)	1473	210	65	44	1
Anti-RBD IgG (BAU/mL)	817	205	66	45	n.d.
Anti-S1 IgG (BAU/mL)	766	246	50	46	n.d.
Anti-Spike IgG (BAU/mL)	832	241	86	53	n.d.
Anti-N IgG (BAU/mL)	713	295	146	12	n.d.

**Table 2:** ELISA titration endpoints from binding assay of NIBSC standard plasma (left column) to SARS-CoV-2 S and N proteins. Plasma was diluted until endpoint binding was achieved. Titres are shown as the first dilution of IgG and IgA to reach <0.2 OD450 nm.

	IgG Spike	IgA Spike	IgG Nucleocapsid	IgA Nucleocapsid
High (20/150)	1:25,600	1:800	>1:51,200	1:800
Mid (20/148)	1:6400	1:200	1:25,600	1:1600
Low S, High N (20/140)	1:1600	1:100	1:12,800	1:100
Low (20/140)	1:800	1:100	1:800	1:100
Negative (20/142)	1:100	1:100	1:200	1:100

## Data Availability

Data are available upon request from the corresponding author.
